# CircASH2L Promotes Ovarian Cancer Tumorigenesis, Angiogenesis, and Lymphangiogenesis by Regulating the miR-665/VEGFA Axis as a Competing Endogenous RNA

**DOI:** 10.3389/fcell.2020.595585

**Published:** 2020-11-19

**Authors:** Jinxin Chen, Xiaocen Li, Lu Yang, Mengmeng Li, Ye Zhang, Jingru Zhang

**Affiliations:** ^1^Department of Gynecology, Cancer Hospital of China Medical University, Liaoning Cancer Hospital & Institute, Shenyang, China; ^2^Department of Graduate School, Dalian Medical University, Dalian, China; ^3^Medical Oncology Department of Gastrointestinal Cancer, Cancer Hospital of China Medical University, Liaoning Cancer Hospital & Institute, Shenyang, China; ^4^Department of Radiation Oncology, Cancer Hospital of China Medical University, Liaoning Cancer Hospital & Institute, Shenyang, China

**Keywords:** *circASH2L*, ceRNA, VEGFA, ovarian cancer, tumorigenesis, angiogenesis

## Abstract

Ovarian cancer is the leading cause of gynecologic cancer-related deaths. Emerging research has revealed a close relationship between circular RNAs (circRNAs) and ovarian cancer development, metastasis, and prognosis. The objective of our research was to further explore the relationship between circASH2L and ovarian cancer. Quantitative real-time polymerase chain reaction was used to detect the differential expression of circRNAs between normal ovaries and ovarian cancer tissues. The impact of circASH2L on the proliferation, invasion, and tumorigenicity of ovarian cancer cells was evaluated using gain- and loss-of-function experiments. The molecular mechanisms of circASH2L function were investigated using bioinformatics analysis, RNA fluorescence *in situ* hybridization, western blots, and dual-luciferase reporter assays. The results showed that circASH2L was remarkably upregulated in ovarian cancer. The invasion and growth of ovarian cancer cells were suppressed by circASH2L knockdown *in vitro*, and downregulation of circASH2L restrained both angiogenesis and lymphangiogenesis of tumor xenografts *in vivo*. Furthermore, circASH2L was mostly distributed in the cytoplasm, where it competes with vascular endothelial growth factor A (VEGFA) for binding to miR-665. These findings indicate that circASH2L has an oncogenic function in ovarian cancer. In conclusion, circASH2L plays a critical role in regulating ovarian cancer cell tumorigenesis, angiogenesis, and lymphangiogenesis through the miR-665/VEGFA axis and, therefore, is a possible candidate target for ovarian cancer treatment.

## Introduction

Ovarian carcinoma is the sixth most commonly diagnosed cancer among women in the world, as well as the second most frequent gynecologic malignancy and the most fatal tumor of the human female reproductive system (Szajnik et al., 2016; Zhan et al., 2018). Over 75% of affected women are diagnosed at advanced stages of the disease (Zhang L. et al., 2019). Less than one third of late-stage patients survive 5 years after diagnosis (Bonnefond et al., 2015; Ricci et al., 2016). Most patients develop metastatic disease after surgery and intensive platinum–taxane chemotherapy (Hollis et al., 2019; Rose et al., 2019). Further investigation of the molecular mechanism underlying the progression of ovarian cancer for the identification of novel therapeutic targets is urgently needed.

Circular RNA (circRNA) is a type of endogenous RNA that forms a covalently closed continuous loop structure without a 5′-cap or a 3′-poly A tail (Wang et al., 2019c; Wei et al., 2020). Owing to its structure, circRNA is more stable than linear RNA and can be resistant to exonucleolytic RNA decay (Si et al., 2019; Liu T. et al., 2020). CircRNA is involved in the regulation of many diseases by interacting with disease-associated microRNA (miRNA) as a competing endogenous RNA (ceRNA) (Wang et al., 2018; Zhang Y. et al., 2019; Li H. et al., 2020). Furthermore, circRNAs may be good biological markers for diagnosis and prognosis of disease, and possibly successful therapeutic targets (Zhuang et al., 2017; Fu et al., 2018; Ye et al., 2019).

Vascular endothelial growth factors (VEGFs), including VEGFA, VEGFB, VEGFC, VEGFD, VEGFE, and placental growth factor, are very important mediators of lymphangiogenesis and angiogenesis during tumor development (Pang et al., 2017; Mesquita et al., 2018; Yang et al., 2018). VEGFs and their receptors (VEGFRs) are targets of anti-angiogenic cancer therapy (Tugues et al., 2011). All members of the VEGF family are characterized by the presence of a common homology domain and are further composed of isoforms with various functions in the human body. VEGFs generally bind to three tyrosine kinase receptors: VEGFR-1, VEGFR-2 [kinase domain receptor (KDR)], and VEGFR-3 [Fms-related tyrosine kinase 4 (FLT4)]. VEGFR-1 and KDR are mostly distributed on the surface of tumor blood vessel endothelium and regulate the generation of tumor blood vessels. FLT4 is mostly distributed on the surface of the lymphatic endothelium and regulates the generation of tumor lymphangion (de Santa Pau et al., 2009; Detoraki et al., 2009; Zhang et al., 2014). Several studies have shown that VEGFA is involved in the pathophysiology of ovarian cancer. For example, Jang et al. (2017) reported that VEGFA activates an epigenetic pathway that upregulates ovarian cancer-initiating cells. Wang and his colleagues (Wang et al., 2019a) reported that the tumorigenicity and progression of ovarian cancer were promoted by circRhoC, which functions as a miR-302e sponge to positively regulate VEGFA.

In this study, the impact of *circASH2L* on the proliferation, invasion, lymphangiogenesis, and angiogenesis of ovarian cancer was analyzed both *in vivo* and *in vitro.* We found that *circASH2L* competes with VEGFA for binding to miR-665 to play an oncogenic role in ovarian cancer.

## Materials and Methods

### Clinical Specimens and Cell Lines

Fifty ovarian cancer tissue samples and their corresponding adjacent normal tissues were obtained from the Liaoning Cancer Hospital & Institute. None of the patients underwent radiotherapy or chemotherapy before surgery. The relationship between circASH2L expression and clinical features (*n* = 50) is shown in Table 1. Our study was approved by the Ethics Committee of Liaoning Cancer Hospital & Institute. Each patient provided written informed consent.

**TABLE 1:** Association of *circASH2L* expression with clinicopathological features of ovarian cancer.

Feathers	Number	High	Low	*P* value
All cases	50	25	25	
Age(years)				0.5709
<50	23	10	13	
≥50	27	15	12	
Tumor size (cm)				**0.0209**
<4	21	6	15	
≥4	29	19	10	
Lymph node metastasis				**0.0157**
Negative	17	4	13	
Positive	33	21	12	
FIGO stage				**0.0101**
I/II	26	8	18	
III/IV	24	17	7	

*Total data from 50 tumor tissues of ovarian cancer patients were analyzed. For the expression of *circASH2L* was assayed by qRT-PCR, the median expression level was used as the cutoff. Data were analyzed by chi-squared test and Fisher’s exact test. *P*-value in bold indicates statistically significant.*

Human ovarian cancer cell lines (A2780, TOV112D, OVCAR-3, and SKOV3) and a normal human ovarian cell line (ISOE80) were obtained from the American Type Culture Collection (Manassas, VA, United States).

### Lentivirus and Short Hairpin (sh)RNA Transfection

We ordered the lentiviral vector system, shRNAs, and empty vectors from GeneChem (Shanghai, China). Oligonucleotides for mimics, inhibitors, and negative control were obtained from RiboBio (Guangzhou, China). The miR-665 mimics and inhibitor were purchased from the same company. After culturing for 1 day, cells were transfected with plasmids. After 2 days, the cells were harvested, and their RNA was extracted.

### Quantitative Real-Time Polymerase Chain Reaction (qRT-PCR)

After centrifugation at 4°C, isopropanol precipitates were harvested at 20–25°C from the upper aqueous phase, rinsed, and dried. Subsequently, we added DEPC-treated water and calculated the concentration of RNA in each sample. RNA was stored at −80°C. We generated cDNA using the OneStep PrimeScript^®^ miRNA cDNA Synthesis Kit (Takara, Dalian, China), per the manufacturer’s instructions. We utilized the SYBR Green I fluorescence method to conduct PCR detection. Primer sequences are provided in Supplementary Table S1. The relative RNA concentrations of the samples were calculated using 2^–ΔΔCt^.

### RNase R Digestion

We added three units of RNase R (Epicenter Biotechnologies, Madison, WI, United States) per 1 μg circASH2L and incubated the mixture at 37°C for 15 min. Next, we conducted qRT-PCR to evaluate the levels of GAPDH and circASH2L.

### Cell Counting Kit-8 Assay

Cell proliferation was assessed using the Cell Counting Kit-8 (Beyotime Institute of Biotechnology, Shanghai, China). Briefly, we seeded cells into 96-well plates and transfected them with appropriate plasmids and controls. We used a microplate reader (Bio-Rad, Hercules, CA, United States) (absorbance wavelength, 450 nm) to assess cell proliferation.

### Ethynyl-2-Deoxyuridine (EdU) Incorporation Assay

Cell proliferation was also assessed using an EdU incorporation assay. Briefly, after transfecting cells with plasmids, 100 μL of 50 μM EdU/well was added and the cells were incubated for 2 h at 37°C. We used fluorescence microscopy to determine proliferation.

### Transwell Invasion Assays

Transwell inserts (8 μm pore size; Costar, Richmond, VA, United States) were employed to determine the potential of cell invasion. Cells (5 × 10^4^/L) were resuspended in 200 μL serum-free medium, then placed into the upper chamber coated with Matrigel (BD Biosciences, Franklin Lakes, NJ, United States). Afterward, 600 μL of complete medium was added to the lower chamber. After 48 h at 37°C, cells that remained on the upper filter surface were removed. After being fixed with formaldehyde and stained with crystal violet, cells that invaded the bottom surface of the filter were counted.

### Western Blotting

We prepared, electrophoresed, and transferred total cell lysates onto nitrocellulose membranes. They were then blocked and incubated with primary antibodies against VEGFA (ab52917), KDR (ab5473), and FLT4 (ab154079) at 4°C overnight. Subsequently, they were incubated with the corresponding secondary antibodies (all antibodies were from Abcam, Shanghai, China).

### RNA Fluorescence *in situ* Hybridization (FISH)

RNA FISH (Ribo^TM^ Fluorescent *in situ* Hybridization Kit, RiboBio) was used to assess the subcellular localization of circASH2L RNA in ovarian cancer cells. A circASH2L probe labeled with the FAM fluorescent dye was used. We carried out RNA FISH and captured the images.

### Dual-Luciferase Reporter Assays

Before cloning into the pRL-TK plasmid vector (Promega, WI, United States), wild-type circASH2L, or wild-type VEGFA, with miR-665 binding sites or mutant sequences with deleted binding sites, were established and amplified. Subsequently, HEK-293T cells were seeded and co-transfected with the luciferase plasmids (0.1 μg/well), and miR-665 mimics or controls. Two days later, firefly and Renilla luciferase activities were measured.

### Tumor Xenograft Implantation Into Nude Mice

Four-week-old female athymic BALB/c nude mice were used. Animal studies were implemented under the guidelines of the Use Committee for Animal Care. We transfected SKOV3 cells (1 × 10^7^/ml) with sh-circASH2L or sh-non-sense control (NC), then injected them subcutaneously into the right flanks of the mice. Tumors were measured every 7 days post-injection. Tumor volumes were calculated (as a rotational ellipsoid) using length × width^2^ × 0.5. Four weeks later, the mice were anesthetized with 40 mg sodium pentobarbital and then sacrificed by 10% formalin perfusion fixation of the central nervous system. Death was confirmed by complete stoppage of the heartbeat and breathing, as well as disappearance of the foot withdrawal reflex. Tumor tissues were isolated and weighed. Tumors were collected for immunohistochemistry and western blot analyses.

### Tube Formation Assay

A tube formation assay was used to determine the *in vitro* angiogenic activity of human umbilical vein endothelial cells (HUVECs). A significant angiogenic property of HUVECs is capillary tube formation on Matrigel. After transfection, HUVECs were serum-starved in endothelial basal medium (United States) with 0.2% bovine serum albumin for 24 h. After harvesting, HUVECs (8 × 10^4^ cells) were seeded into a 12-well plate coated with Matrigel (BD Biosciences). Using a computer-assisted microscope (Nikon), we observed tube formation after 8 h of incubation. Tube formation was defined as a structure with a length four times its width. We selected 10 random microscopic fields and took images of the tube morphology at 100 × magnification. Using LAS software (Leica), we determined the number of tubes.

### Immunohistochemistry

Paraffin-embedded formalin-fixed tissues were cut into 4-μm-thick sections and processed for immunohistochemistry using rabbit anti-mouse LYVE-1 and rat anti-mouse CD34 (Abcam). The Poly-Horseradish Peroxidase Detection System and 3,3′-diaminobenzidine (Zhongshan Biotechnology, Beijing, China) were used to detect immunoreactivity.

### Statistical Analyses

Student’s t or the χ2 test were used to assess the statistical significance of differences using data from 3 independent experiments. Data are presented as means ± standard deviation. Statistical significance was set at *p* < 0.05. All statistical tests were performed using SPSS version 19.0 software (IBM, Chicago, IL, United States).

## Results

### CircASH2L Expression Is Elevated in Ovarian Cancer Tissues and Cell Lines

**FIGURE 1:**
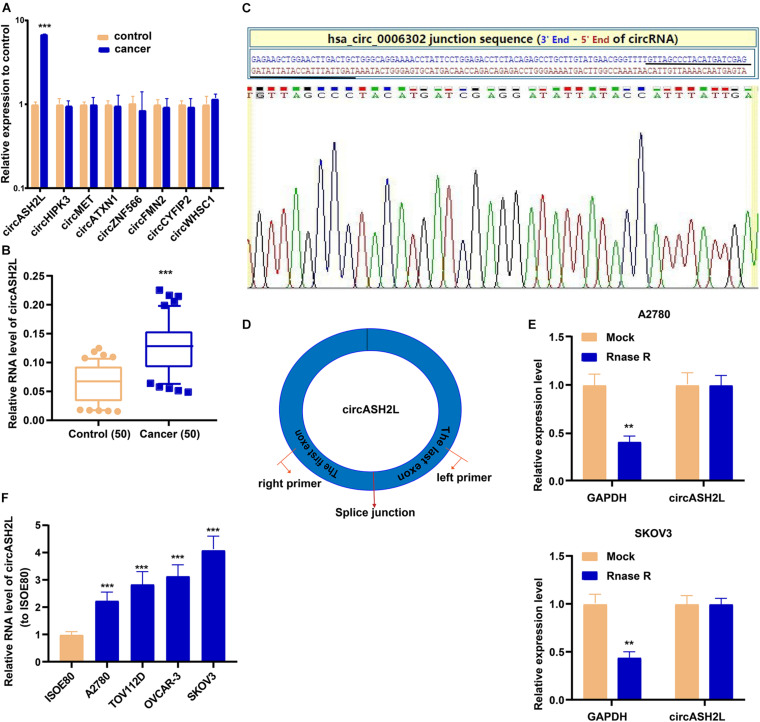
High expression of *circASH2L* in ovarian cancer. **(A)** 8 different circRNAs including *circASH2L* in ovarian cancer tissue and paired non-tumor tissue counterparts. **(B)** *circASH2L* was elevated in 50 pairs ovarian cancer tissues. **(C)** Sanger sequencing of *circASH2L*. **(D)** Schematic outlining the details of *circASH2L* and its primer-designing details. **(E)** RNase R digestion was used to verify the circular nature of *circASH2L*. **(F)** Relative expression of *circASH2L* in ovarian cancer cell lines and ISOE80 cells by qRT-PCR. Data represent the mean ± SD. **p < 0.01; ***p < 0.001.

Increasing evidence has shown that circRNAs, such as circASH2L (Chen et al., 2019), circHIPK3 (Wang et al., 2019b), circMET (Pei et al., 2020), circATXN1 (Liu X. et al., 2020), circZNF566 (Li S. et al., 2020), circFMN2 (Shan et al., 2020), circCYFIP2 (Lin et al., 2020), and circWHSC1 (Zong et al., 2019), have essential functions in cancer biology and are potential biomarkers and treatment targets. We determined the expression of the eight star circRNAs in ovarian cancer and found that circASH2L was the only one expressed differently in our samples (Figure 1A). The qRT-PCR results of 50 pairs of ovarian cancer samples and corresponding non-tumor tissue showed that circASH2L was obviously increased in ovarian cancer tissues (Figure 1B). We also confirmed that the circASH2L sequence, amplified by the primer, matched the sequence of Circbase that was derived from Sanger sequencing (Figure 1C). The details of circASH2L and its primer-designing are outlined in Figure 1D. Total RNA was then treated with RNase R to verify the circular nature of circASH2L. The data revealed that circASH2L was indeed a circRNA and could not be digested by RNase R (Figure 1E). As shown in Figure 1F, circASH2L expression was significantly higher in ovarian cancer cell lines (A2780, TOV112D, OVCAR-3, and SKOV3) than in normal human cells (ISOE80).

### CircASH2L Enhances the Invasion and Proliferation of Ovarian Cancer Cells

**FIGURE 2:**
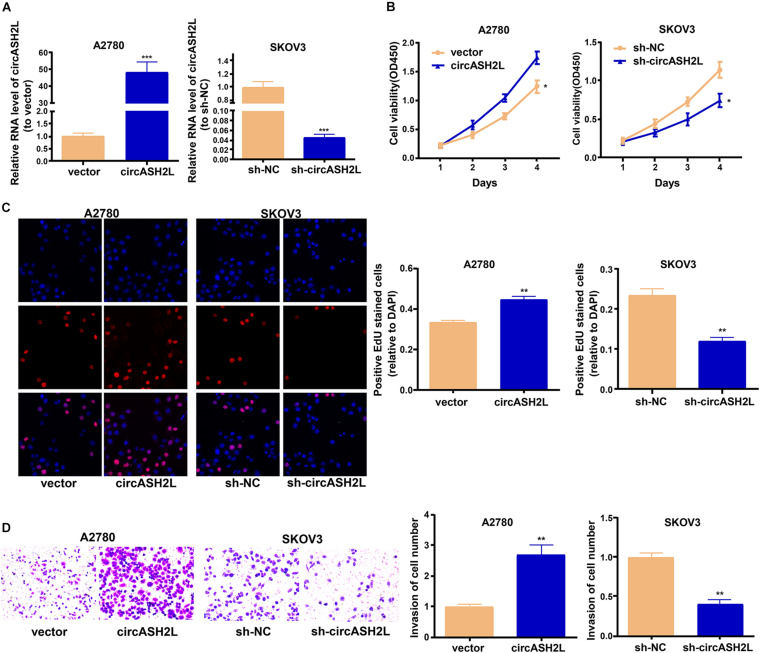
*CircASH2L* promotes the growth and invasion of ovarian cancer cells. **(A)** Transfection effectiveness of *circASH2L* shRNAs in SKOV3 cells and *circASH2L* overexpression in A2780 cells. **(B)** The proliferation of ovarian cancer cells was detected by CCK-8 assay. **(C)** The proliferation of ovarian cancer cells was detected by EdU assay. **(D)** The invasion of ovarian cancer cells was detected by transwell assay. Data represent the mean ± SD of 3 independent experiments; *p < 0.05; **p < 0.01; ***p < 0.001.

We investigated whether circASH2L modulated the development of ovarian cancer cells. First, we tested the transfection efficiency of sh-circASH2L in SKOV3 cells, and circASH2L overexpression in A2780 and ISOE80 cells (Figure 2A and Supplementary Figure S1A). We then used CCK-8, EdU, and transwell assays to assess the abilities of these cell to proliferate and invade. The proliferation of SKOV3 cells was suppressed by circASH2L knockdown, and its overexpression increased the proliferation of A2780 cells (Figures 2B,C). Meanwhile, we found that overexpression of circASH2L promotes ISOE80 cell proliferation (Supplementary Figure S1B). In addition, circASH2L knockdown inhibited the invasion of SKOV3 cells, while its overexpression enhanced the invasion of A2780 cells (Figure 2D). Altogether, these results demonstrated that circASH2L enhanced ovarian cancer cell proliferation and invasion *in vitro*.

### CircASH2L Promotes VEGFA Expression by Binding to miR-665

**FIGURE 3:**
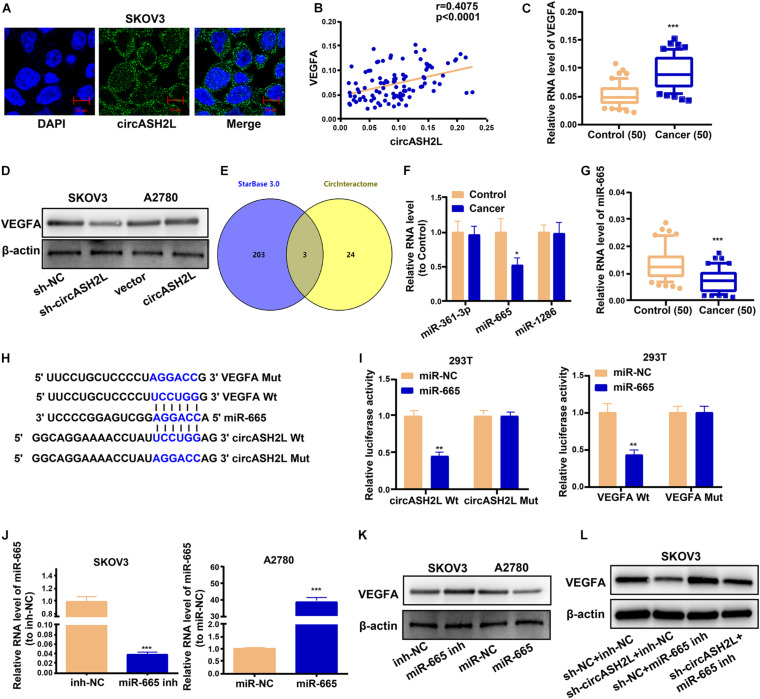
*CircASH2L* enhances *VEGFA* expression by binding to miR-665. **(A)** Representative images of the subcellular localization of *circASH2L* by RNA-FISH in SKOV3 cells. **(B)** Evaluating the correlation of *circASH2L* and *VEGFA* expression in ovarian cancer tissues by Pearson’s correlation coefficient. **(C)** The expression level of *VEGFA* in ovarian cancer tissues. **(D)** The effect of *circASH2L* knockdown or overexpression on the expression of *VEGFA* in ovarian cancer cells by western blotting. **(E)** Bioinformatics analysis with starBase 3.0 and Circinteractome. **(F)** The level of miR-361-3p, miR-665, and miR-1286 in ovarian cancer tissues. **(G)** The level of miR-665 in ovarian cancer tissues and corresponding non-tumor tissues. **(H,I)** The binding sites and dual-luciferase reporter assay. **(J)** The transfection efficiency of miR-665 mimics in A2780 cells and miR-665 inhibitor in SKOV3 cells. **(K)** The effect of miR-665 on the expression of *VEGFA* in ovarian cancer cells. **(L)** The effect of miR-665 inhibitor and sh-*circASH2L* on the expression of *VEGFA* in ovarian cancer cells by western blotting. Data represent the mean ± SD of 3 independent experiments. *p < 0.05; **p < 0.01; ***p < 0.001.

We investigated the mechanism of circASH2L functions in ovarian cancer. RNA FISH showed that most circASH2L was localized to the cytoplasm, whereas little was found in the nucleus (Figure 3A).

Previous studies found that VEGFA played a role in the pathophysiological process of ovarian cancer (Shathasivam et al., 2017; Lu et al., 2018). We investigated whether circASH2L affected ovarian cancer by regulating VEGFA expression. We used qRT-PCR and western blotting assays to characterize the molecular mechanisms of circASH2L-mediated biological processes. The levels of circASH2L were positively correlated with VEGFA expression in ovarian cancer samples (Figure 3B). QRT-PCR results of 50 patients showed that VEGFA mRNA expression was markedly higher in ovarian cancer samples than in paired non-tumor tissue (Figure 3C). Western blotting indicated that knockdown of circASH2L suppressed VEGFA protein expression and increased circASH2L expression promoted VEGFA expression (Figure 3D).

Bioinformatics analyses were performed using starBase 3.0 and Circinteractome, and revealed the intersection. Specifically, miR-361-3p, miR-665, and miR-1286 had putative binding sites with both circASH2L and VEGFA (Figure 3E). Using qRT-PCR, levels of the 3 miRNAs were determined in ovarian cancer tissues. Only miR-665 was differentially expressed between cancer and normal tissues, and its expression in cancer tissues was low (Figure 3F). We also used 50 pairs of tissues from ovarian cancer patients to determine that miR-665 was expressed at remarkably lower levels in ovarian cancer than paired non-tumor tissues (Figure 3G). The relative expression levels of circASH2L, miR-665, and VEGFA in these paired tissues are shown in Supplementary Table S2.

Bioinformatics evaluation revealed that the miR-665 seed sequence was complementary to sequences in the 3′-untranslated regions of both VEGFA and circASH2L (Figure 3H). The results of the dual-luciferase reporter assay showed that luciferase activity in 293T cells was inhibited both the VEGFA Wt + miR-665 mimic and the circASH2L Wt + miR-665 mimic groups, while luciferase activity was not affected in the VEGFA Mut + miR-665 mimic or the circASH2L Mut + miR-665 mimic groups (Figure 3I). The transfection efficiency of the miR-665 mimic in A2780 cells, and the miR-665 inhibitor in SKOV3 cells, is shown in Figure 3J. The miR-665 mimic decreased the VEGFA protein level, while the miR-665 inhibitor increased this level (Figure 3K). Knocking down miR-665 reversed the inhibition of VEGFA induced by knockdown of circASH2L (Figure 3L). These data suggested that circASH2L could bind to miR-665 to regulate VEGFA expression through a ceRNA mechanism.

### Overexpression of VEGFA Reverses the Effects of sh-circASH2L on Ovarian Cancer Cells

**FIGURE 4:**
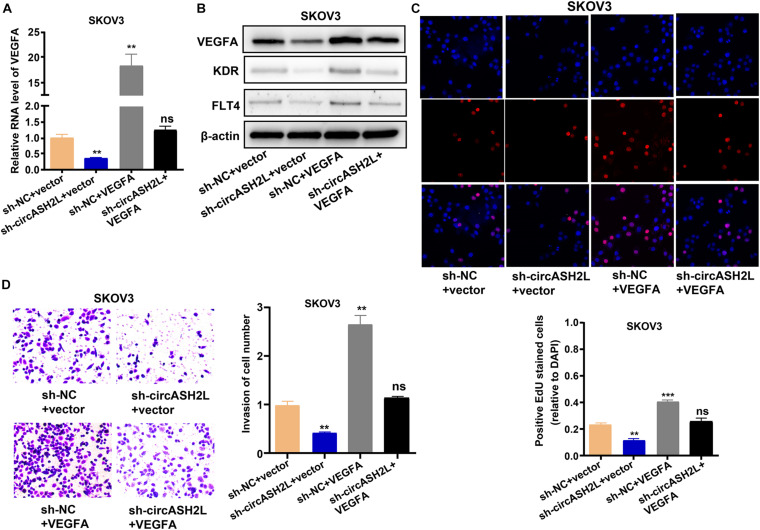
*VEGFA* overexpression reverses the *circASH2L* knockdown-induced inhibition of proliferation and invasion of ovarian cancer cells. **(A)** The effect of sh-*circASH2L* and *VEGFA* on the RNA level of *VEGFA*. **(B)** The effect of sh-*circASH2L* and *VEGFA* on the protein level of *VEGFA*, KDR, and FLT4. **(C)** The effect of sh-*circASH2L* and *VEGFA* on the cell proliferation in SKOV3 cell line. **(D)** The effect of sh-*circASH2L* and *VEGFA* on the cell invasion in SKOV3 cell line. Data represent the mean ± SD of 3 independent experiments; **p < 0.01, ***p < 0.001, ns: no statistical significance.

We next assessed whether circASH2L affected the proliferation and invasion of ovarian cancer cells through a VEGFA-dependent mechanism. VEGFA overexpression significantly reversed the circASH2L knockdown-induced reduction in SKOV3 cells (Figures 4A,B). At the same time, VEGFA overexpression reversed the circASH2L knockdown-induced reduction of KDR and FLT4 expression in SKOV3 cells (Figure 4B). Moreover, VEGFA overexpression significantly reversed the inhibition of proliferation and invasion induced by circASH2L knockdown in SKOV3 cells (Figures 4C,D). These data demonstrated that circASH2L affected the growth and invasion of ovarian cancer cells in a VEGFA-dependent manner.

### Knocking Down CircASH2L Curbs the Growth, Lymphangiogenesis, and Angiogenesis of Tumors in a Mouse Model of Ovarian Cancer

**FIGURE 5:**
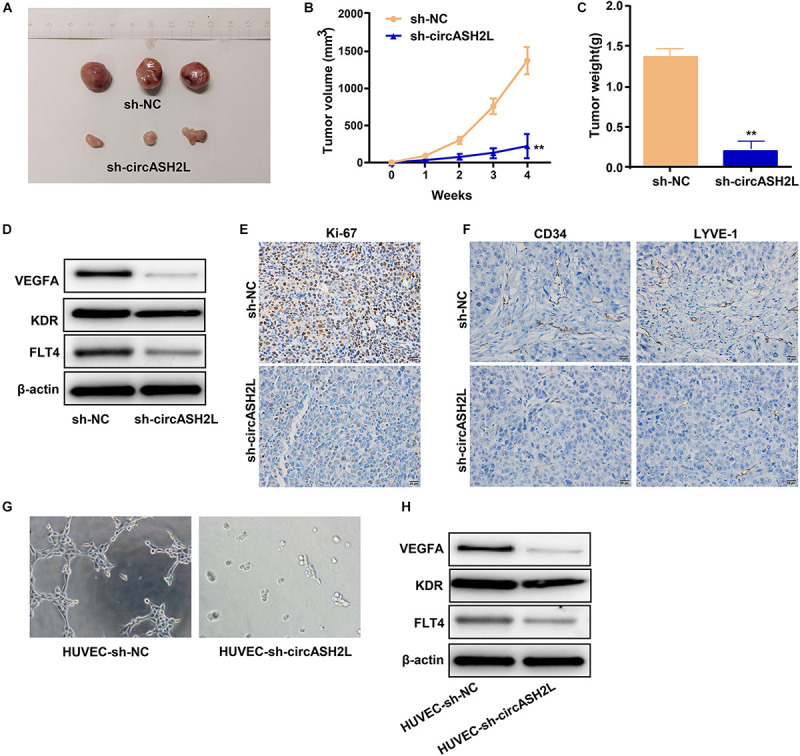
Sh-*circASH2L* hinders the tumor growth, lymphangiogenesis, and angiogenesis. **(A)** Xenograft tumors of ovarian cancer. **(B)** Tumor volumes of the sh-*circASH2L* and sh-NC treatment groups. **(C)** Tumor weights. **(D)** *CircASH2L* knockdown reduces *VEGFA*, KDR, and FLT4 expression *in vivo*. **(E)** *CircASH2L* knockdown reduces the expression of Ki67 in the xenograft tumors of ovarian cancer. **(F)** *CircASH2L* knockdown inhibits angiogenesis and lymphangiogenesis *in vivo*. **(G)** The *in vitro* angiogenesis of HUVECs. **(H)** The protein level of *VEGFA*, KDR, and FLT4 in HUVECs after transfection. **p < 0.01.

A xenograft experiment was conducted with SKOV3 cells to investigate the impact of circASH2L shRNA on tumor cell growth *in vivo*. The xenograft tumors are shown in Figure 5A. Mice treated with sh-circASH2L exhibited decreased tumor volume and weight compared to those treated with sh-NC (Figures 5B,C). Western blot results indicated that sh-circASH2L decreased the protein levels of VEGFA, KDR, and FLT4 (Figure 5D). In sh-circASH2L-treated mice, the Ki-67 level was significantly reduced compared to that in the sh-NC group, as indicated by immunohistochemical staining (Figure 5E).

To analyze lymphangiogenesis and angiogenesis of tumors, samples were assessed by immunohistochemical staining with anti-LYVE-1 and anti-CD34 antibodies. Compared with sh-NC, sh-circASH2L-treated groups showed remarkable reductions in blood vessels and lymphangions (Figure 5F).

We also investigated whether the downregulation VEGFA induced by sh-circASH2L affected tube formation. Sh-NC-transfected HUVECs formed well-organized capillary-like structures (Figure 5G). However, tube-forming activity was significantly weakened in the sh-circASH2L group (Figure 5G), similar to the KDR and FLT4 small interfering (si)RNA transfection groups (Supplementary Figures S2A–D). Finally, we examined the protein levels of VEGFA, FLT4, and KDR in HUVECs after transfection. Western blots showed that the VEGFA, FLT4, and KDR levels in sh-circASH2L-transfected HUVECs were significantly reduced compared to the sh-NC-transfected HUVECs (Figure 5H).

These outcomes indicated that knockdown of circASH2L remarkably suppressed tumor progression *in vivo*, and sh-circASH2L appears to regulate tumorigenesis by inhibiting VEGFA-mediated lymphangiogenesis and angiogenesis.

## Discussion

CircRNA research has been increasing in recent years, and there is substantial evidence to show that circRNA is a tumor-related factor that regulates gene expression in different cells. Therefore, the expression eight circRNAs that were reported to be closely related to cancers was assessed in ovarian cancer and paired non-tumor tissues by qRT-PCR. CircASH2L was selected as the research object because it was highly expressed in tumor compared to normal tissues. Cell function assays were performed to verify circASH2L functions in ovarian cancer, and showed that it functioned as an oncogene, enhancing the proliferation and invasion of ovarian cancer cells.

Some studies have shown that VEGFA promotes tumor cell proliferation and angiogenesis (Guan et al., 2019), and VEGFA is closely related to ovarian cancer (Sopo et al., 2019). We investigated whether circASH2L played a role in ovarian cancer by regulating VEGFA. In this study, the qRT-PCR, western blot assays, bioinformatics analyses, and the dual-luciferase reporter assay showed that circASH2L competes with VEGFA for binding to miR-665, thereby playing an oncogenic role in ovarian cancer. Finally, we performed a xenograft experiment and *in vitro* angiogenesis assays, and found that sh-circASH2L inhibited tumor growth, VEGFA-mediated angiogenesis, and lymphangiogenesis *in vivo* and *in vitro*. Jang et al. (2017) found that VEGFA stimulated ovarian cancer stem-like cells through Src-DNMT3A-driven miR-128-2 methylation and Bmi1 upregulation. The research of Cybulski et al. (2012) indicated that elevated expression of cyclin I and KDR was likely to provide a proliferative advantage to human epithelial ovarian cancer cells. Meanwhile, KDR silencing by siRNA suppressed epithelial ovarian cancer invasion (Wang et al., 2009). Furthermore, Chen et al. (2019) found that circ-ASH2L promoted tumor progression by sponging miR-34a to regulate Notch1 in pancreatic ductal adenocarcinoma. Whether miR-34a/Notch1 is also regulated by circASH2L in ovarian cancer needs further study.

**FIGURE 6:**
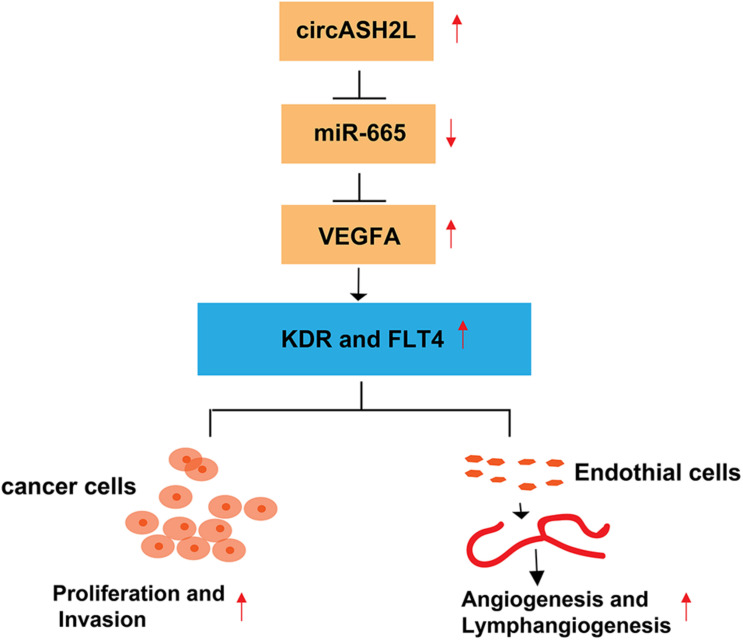
Hypothetical model of the *circASH2L* stimulate roles in ovarian cancer cells and endothelial cells. *CircASH2L* enhances *VEGFA* expression by operating as a miRNA sponge and sequestering miR-665 away from *VEGFA* mRNA. The protein level of *VEGFA*, KDR, and FLT4 increase, thereby, enhancing the proliferation and invasion of ovarian cancer cells and promoting the angiogenesis and lymphangiogenesis.

In conclusion, this research demonstrated that circASH2L is overexpressed in ovarian cancer tissues and cell lines. Regulating the miR-665/VEGFA axis as a ceRNA, circASH2L promotes cell proliferation, invasion, VEGFA-mediated angiogenesis, and lymphangiogenesis to play an oncogenic role in ovarian cancer (Figure 6).

## Data Availability Statement

The original contributions presented in the study are included in the article/Supplementary Material, further inquiries can be directed to the corresponding author.

## Ethics Statement

The animal study was reviewed and approved by the Ethics Committee of Liaoning Cancer Hospital & Institute.

## Author Contributions

JZ made prosperous contributions to conception and design. JC and XL performed the experiments. JC wrote the draft manuscript. LY analyzed the data. ML and YZ made collection of data. All authors made contributions to the examination of the manuscript, and approved the final manuscript for submission.

## Conflict of Interest

The authors declare that the research was conducted in the absence of any commercial or financial relationships that could be construed as a potential conflict of interest.

## Abbreviations

circRNA: circular RNA
miR: microRNA
shRNA: short hairpin RNA
qRT-PCR: quantitative real-time polymerase chain reaction
EdU: ethynyl-2-deoxyuridine
VEGFA: vascular endothelial growth factor A
FISH: fluorescent *in situ* hybridization
ceRNA: competing endogenous RNA.
