# Supplementary material for: CircASH2L Promotes Ovarian Cancer Tumorigenesis, Angiogenesis, and Lymphangiogenesis by Regulating the miR-665/VEGFA Axis as a Competing Endogenous RNA
Source: Front Cell Dev Biol. 2020 Nov 19;8:595585. doi: 10.3389/fcell.2020.595585 (PMC7711110; doi:10.3389/fcell.2020.595585)
Supplement: Supplementary file 1 [file Image_1.PDF]

Figure S1

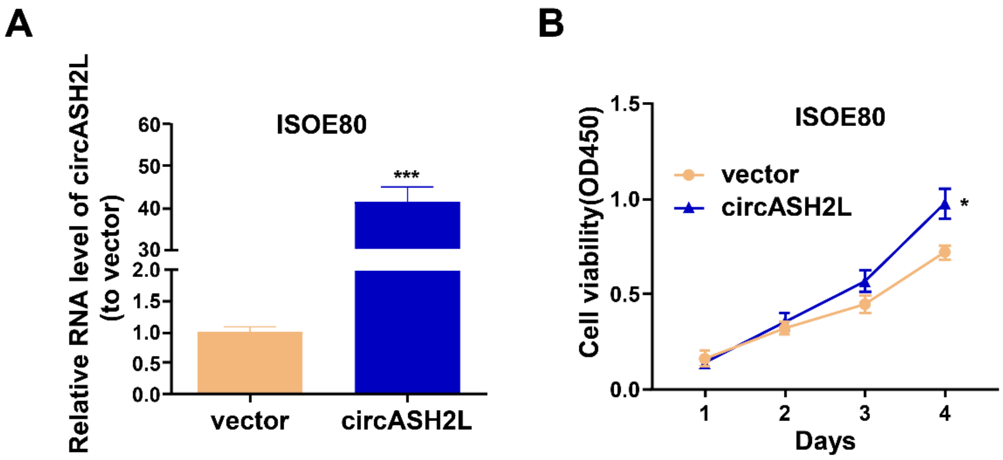

Figure S1. *CircASH2L* enhances cell proliferation of ISOE80 cells.

(A) Transfection effectiveness of *circASH2L* overexpression in ISOE80 cells. (B) The proliferation of ISOE80 cells was detected by CCK-8 assay. Data represent the mean ± SD of 3 independent experiments; $*p < 0.05$; $*p < 0.001$.
