# Supplementary material for: CircASH2L Promotes Ovarian Cancer Tumorigenesis, Angiogenesis, and Lymphangiogenesis by Regulating the miR-665/VEGFA Axis as a Competing Endogenous RNA
Source: Front Cell Dev Biol. 2020 Nov 19;8:595585. doi: 10.3389/fcell.2020.595585 (PMC7711110; doi:10.3389/fcell.2020.595585)
Supplement: Supplementary file 2 [file Image_2.PDF]

Figure S2

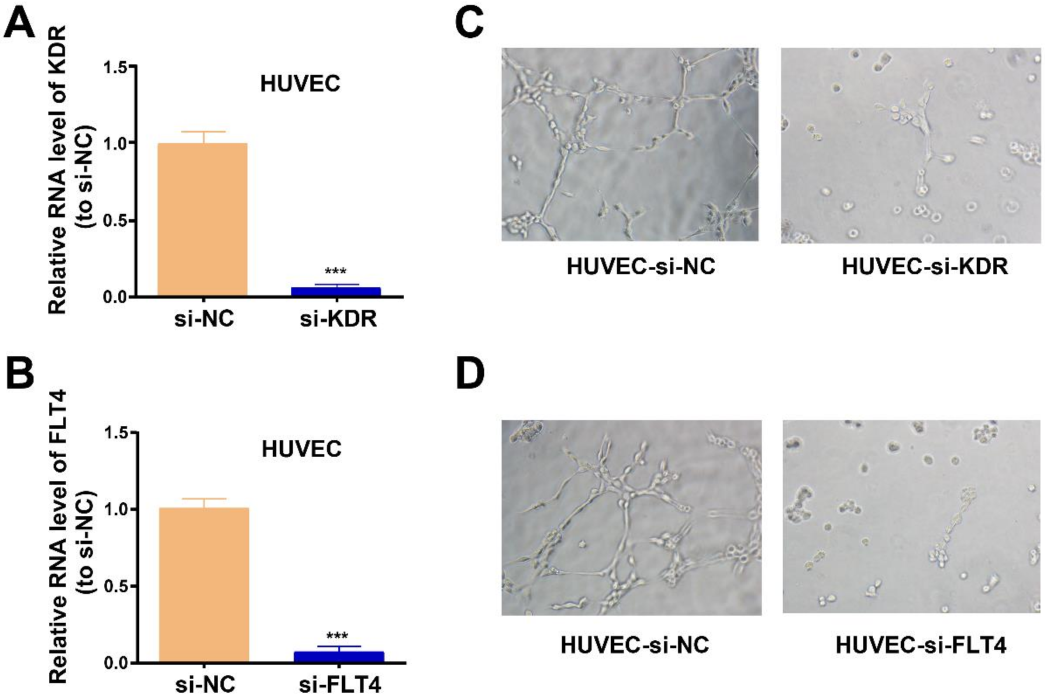

Figure S2. The tube-forming activity was significantly impaired in KDR and FLT4 siRNA transfection group compared to negative controls.

(A) Transfection effectiveness of KDR siRNA in HUVECs. (B) Transfection effectiveness of FLT4 siRNA in HUVECs. (C) The in vitro angiogenesis of HUVECs. (D) The in vitro angiogenesis of HUVECs. Data represent the mean ± SD of 3 independent experiments; ***$p < 0.001$.
