# Supplementary material for: CircASH2L Promotes Ovarian Cancer Tumorigenesis, Angiogenesis, and Lymphangiogenesis by Regulating the miR-665/VEGFA Axis as a Competing Endogenous RNA
Source: Front Cell Dev Biol. 2020 Nov 19;8:595585. doi: 10.3389/fcell.2020.595585 (PMC7711110; doi:10.3389/fcell.2020.595585)
Supplement: Supplementary file 3 [file Table_1.DOCX]

**Table S1.** **Sequences of primers for qRT-PCR**

| **Name** | |  | **Sequence** |
| --- | --- | --- | --- |
| circASH2L | LEFT | | 5’- GCTGGGCAGGAAAACCTATT-3’ |
|  | RIGHT | | 5’- TTTCCCAGGTCTCTGTCTGG-3’ |
| miR-665 | LEFT | | 5'-GGTGAACCAGGAGGCTGAGG-3' |
|  | RIGHT | | 5'-CAGTGCAGGGTCCGAGGTAT-3' |
| VEGFA | LEFT | | 5'- CCTTGCCTTGCTGCTCTAC -3' |
|  | RIGHT | | 5'- TTCTGCCCTCCTCCTTCTG -3' |
| circHIPK3 | LEFT | | 5’- GGCAGCCTTACAGGGTTAAA-3’ |
|  | RIGHT | | 5’- GGGTAGACCAAGACTTGTGAGG-3’ |
| circMET | LEFT | | 5’- TCTTCAACAAGATCGTCAACAAA-3’ |
|  | RIGHT | | 5’- TGGGAAGCATTCTGCTAAAC-3’ |
| circATXN1 | LEFT | | 5’- TCCAGCACCGTAGAGAGGAT-3’ |
|  | RIGHT | | 5’- GTAGGGGATCCAGGCTCTTC-3’ |
| circZNF566 | LEFT | | 5’- TGGCAATGCCTTTAGTCAGA-3’ |
|  | RIGHT | | 5’- TTTCCCACTGGGTTGATTCT-3’ |
| circFMN2 | LEFT | | 5’- TCAGAAACTCCCCAAAAACG-3’ |
|  | RIGHT | | 5’- AGAAGACCCATGGCAATGAT-3’ |
| circCYFIP2 | LEFT | | 5’- CATCCTCTTCTGCCTCCTCA-3’ |
|  | RIGHT | | 5’- GCCCTGATAACCCAGGAGTC-3’ |
| circWHSC1 | LEFT | | 5’- TTGGTGTGGTCCAAAGTGTC-3’ |
|  | RIGHT | | 5’- CAGAAAGGGGACTCTGCTTG-3’ |
| KDR | LEFT | | 5’- GGCCCAATAATCAGAGTGGCA-3’ |
|  | RIGHT | | 5’- CCAGTGTCATTTCCGATCACTTT-3’ |
| FLT4 | LEFT | | 5’- TGCACGAGGTACATGCCAAC-3’ |
|  | RIGHT | | 5’- GCTGCTCAAAGTCTCTCACGAA-3’ |
